# Supplementary material for: Genome-wide identification of DNA methylation QTLs in whole blood highlights pathways for cardiovascular disease
Source: Nat Commun. 2019 Sep 19;10:4267. doi: 10.1038/s41467-019-12228-z (PMC6753136; doi:10.1038/s41467-019-12228-z)
Supplement: Supplementary file 1 — Supplementary Information [file 41467_2019_12228_MOESM1_ESM.pdf]

Genome-wide Identification of DNA Methylation QTLs in Whole Blood Highlights Pathways for Cardiovascular Disease

Huan et al.

Supplementary Figures

Supplementary Tables

Supplementary Figure 1: Enrichment of CpGs with household effects at >0.1 in different genomic regions.

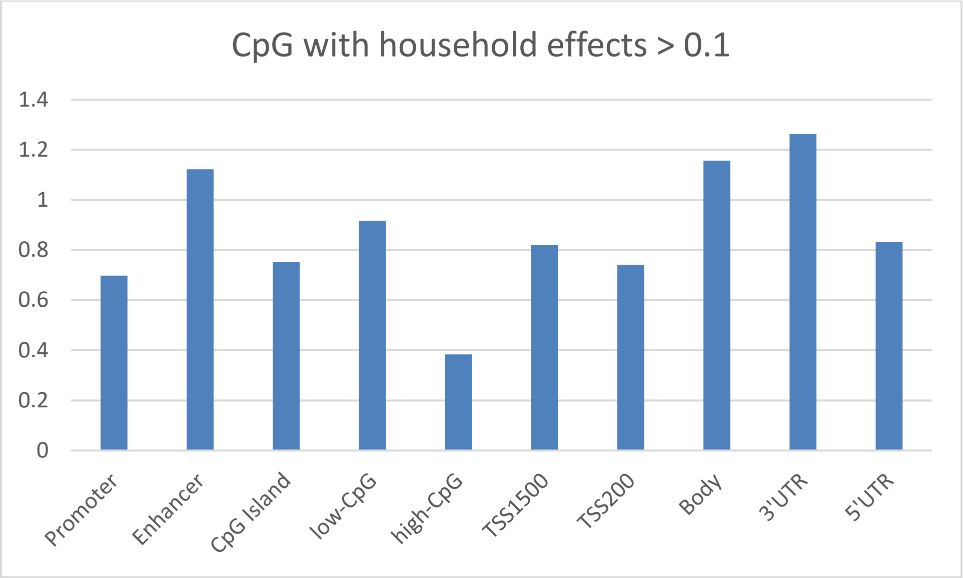

Supplementary Figure 2: Plot distances between *cis*-meQTLs SNPs and CpGs.

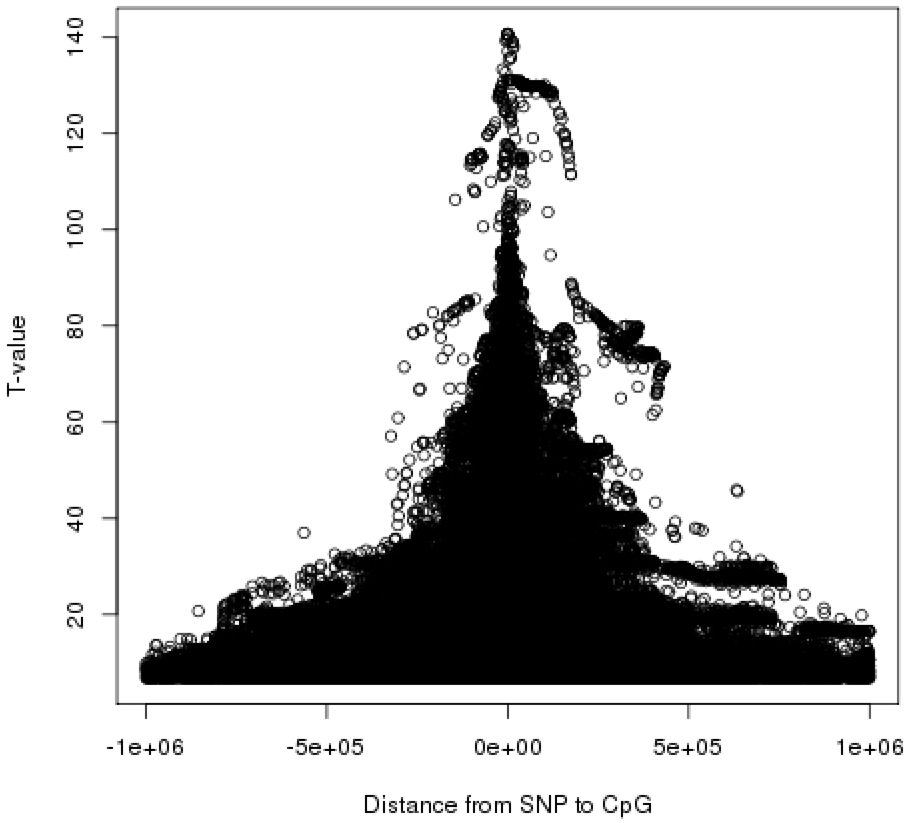

Supplementary Figure 3: Work flow and results summary of the identification and replication of meQTLs.

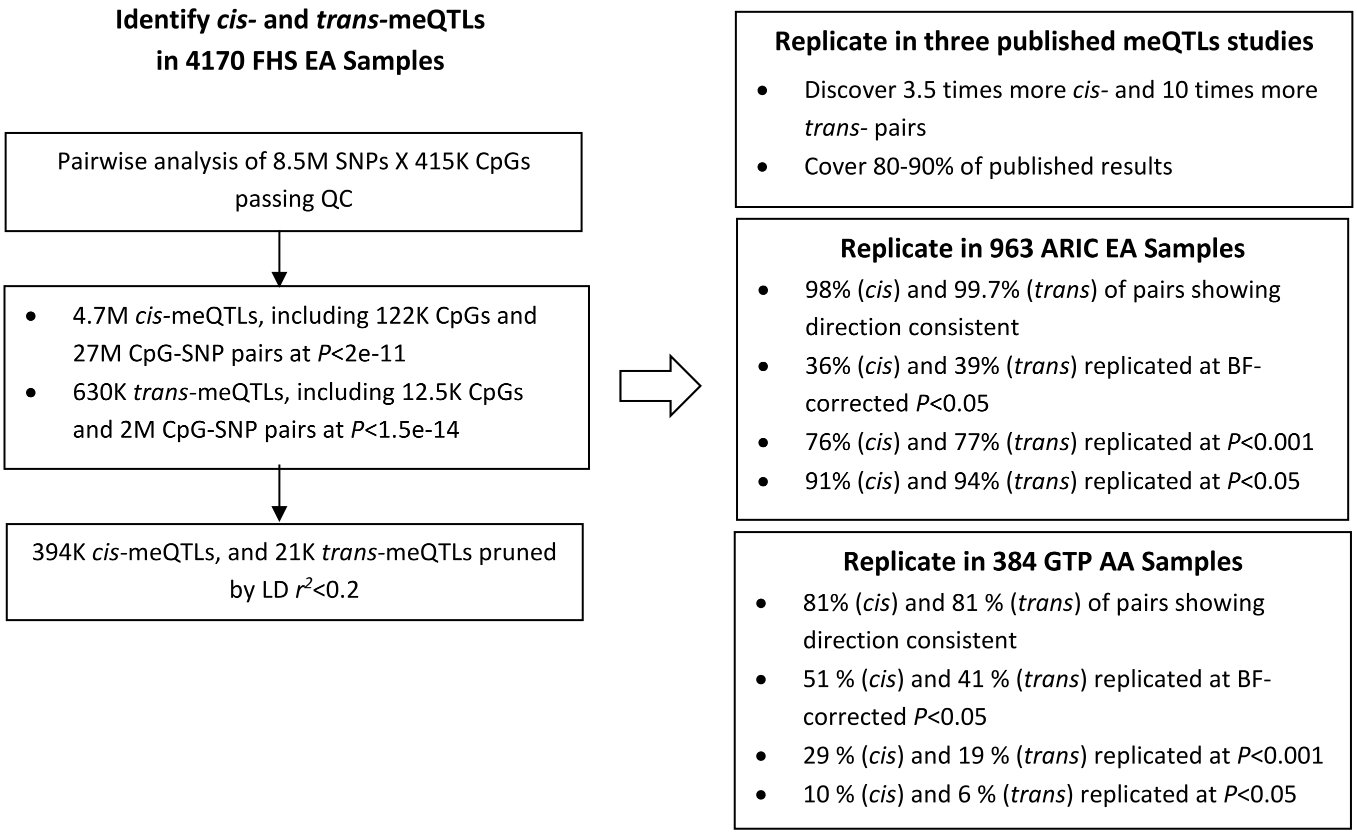

Supplementary Figure 4: QQ-plot of meQTLs.

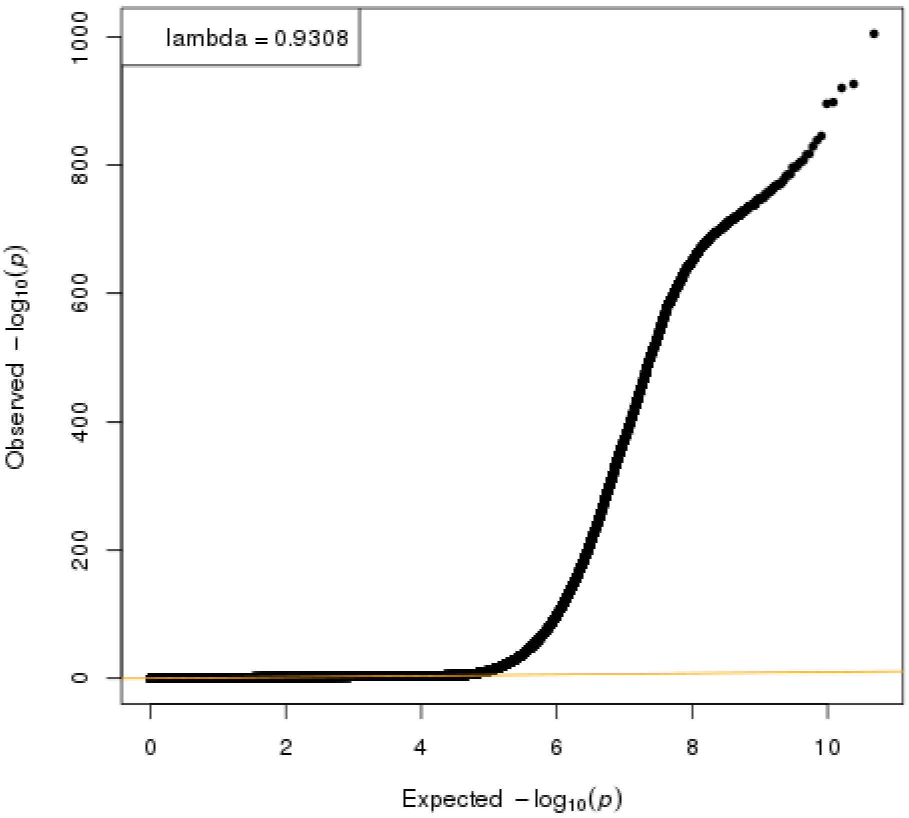

Supplementary Figure 5: Venn Plots of the overlap between meQTLs identified in FHS and published studies. A) *cis*; B) *trans*. **Gaunt-2016:** *Gaunt, T.R., et al., Systematic identification of genetic influences on methylation across the human life course. Genome biology, 2016. 17(1): p. 61.* **Lemire-2015:** *Lemire, M., et al., Long-range epigenetic regulation is conferred by genetic variation located at thousands of independent loci. Nature communications, 2015. 6.* **Bonder-2017:** *Bonder, M.J., et al., Disease variants alter transcription factor levels and methylation of their binding sites. Nature genetics, 2017. 49(1): p. 131.*

A

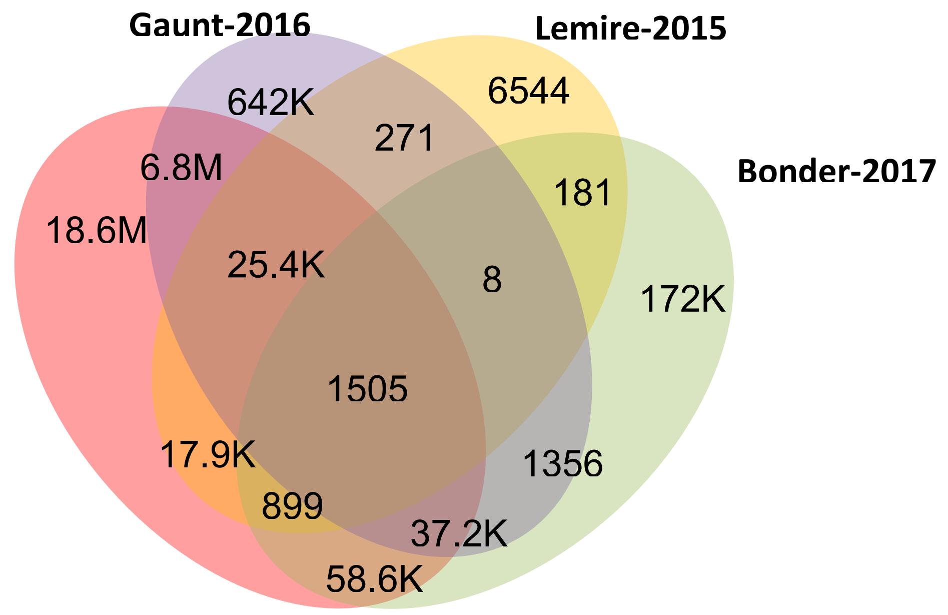

B

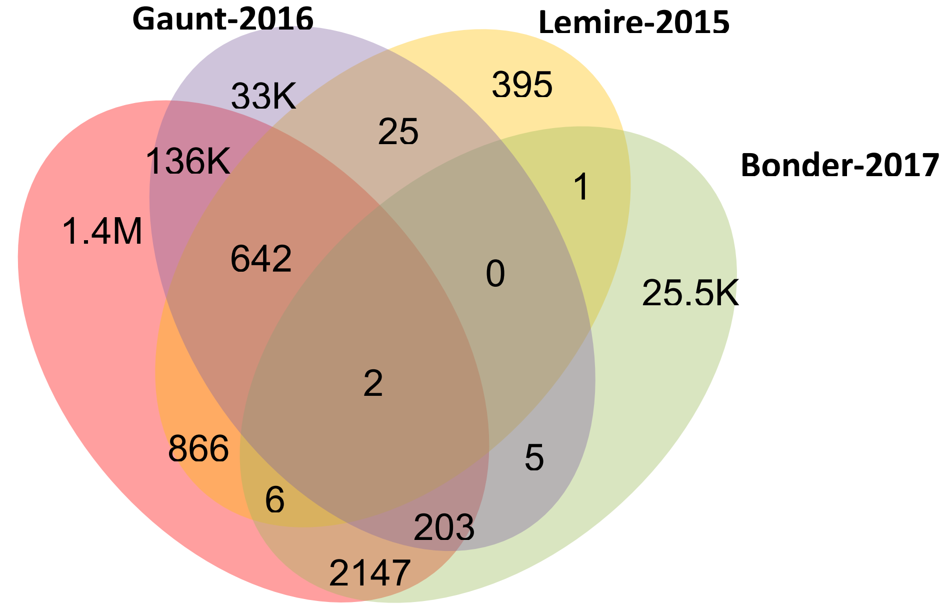

Supplementary Figure 6: Enrichment of meQTL SNPs in different chromatin states in multiple tissues. A) *cis*; B) *trans*.

A

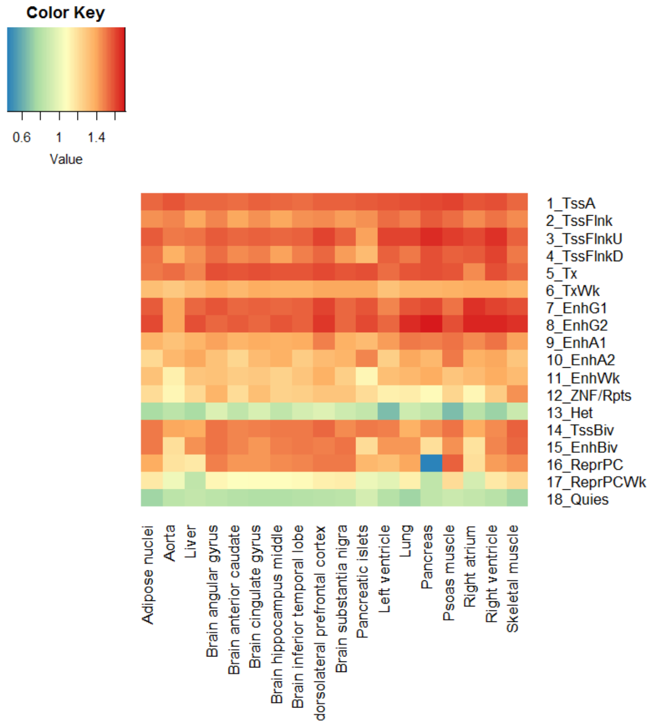

B

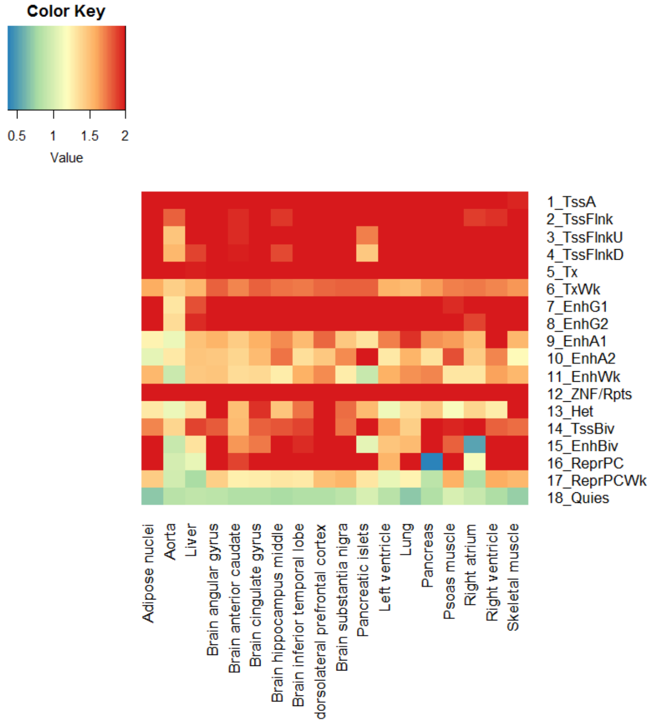

Supplementary Figure 7: MR-Egger regression scatterplots for effects of exposure on outcome. A) cg12555086 on CHD; B) *LIPA* expression on CHD; C) cg06882058 on SBP; D) *SDCCAG8* expression on SBP; Scatter plots demonstrate the relationships of SNP effects on exposure (i.e., CpG or gene expression) and on the outcome (i.e., CHD and SBP). Each black dot in the scatter plots represents a *cis*-meQTL / eQTL SNP, and the red dots shows the independent SNPs pruned by LD r^2<0.01 as IVs, with standard error bars. The lines show the MR-Egger regression lines regressed from all *cis*-meQTLs / eQTLs (black) or independent *cis*-meQTLs / eQTLs (red). The estimated MR-Egger intercepts are non-significantly different from 0 at *P*>0.05.

A

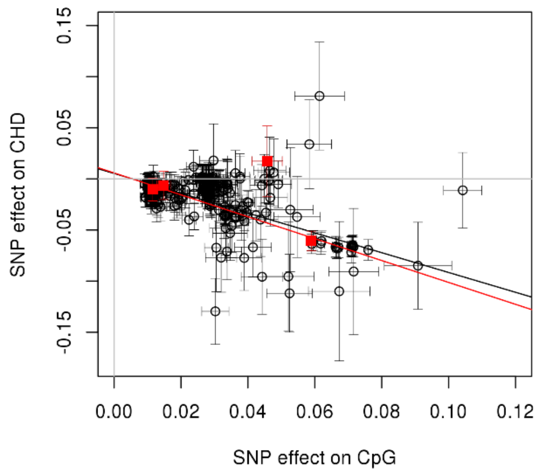

B

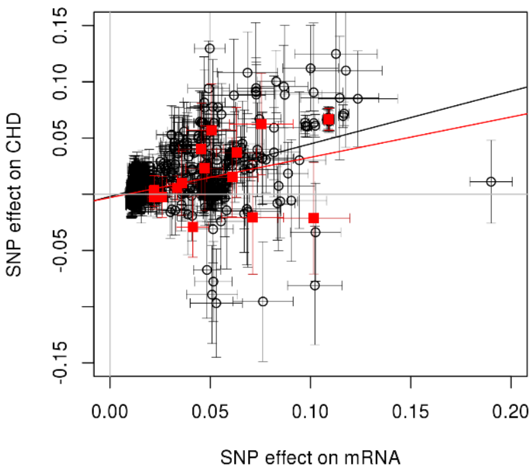

C

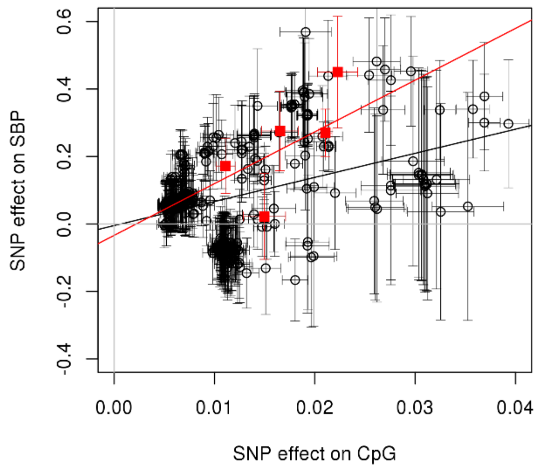

D

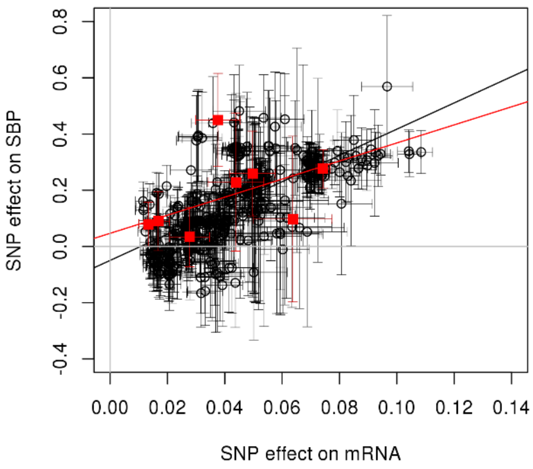

Supplementary Figure 8: 2-D regional plot of *trans*-meQTLs genome-widely. Each dot represents a *trans*-meQTL at *P*<1.5e-14. The dot size is inversely proportional to the *P* values. The largest dot indicates *P*<1e-200. The dots along the diagonal indicate that a SNP and CpG (for *trans*-meQTLs) reside in the same chromosome. *X*-axis: SNP locations in chromosome, *y*-axis: CpG locations in chromosome. The numbers along the *x*-axis and *y*-axis reflect chromosome number.

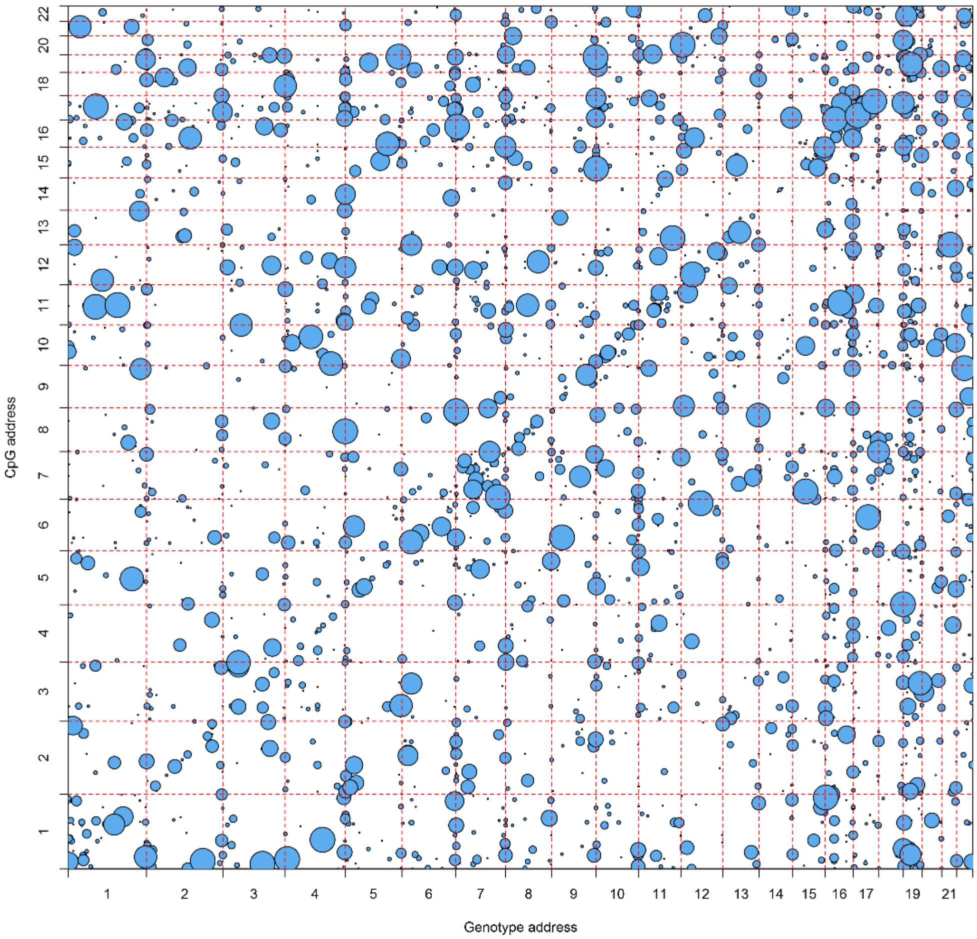

Supplementary Table 1: Characteristics of FHS cohorts.

	Offspring Cohort	Third Generation Cohort
**N**	2648	1522
	**Mean (SD)**	**Mean (SD)**
**Age (year)**	66.37 (8.92)	45.37 (7.85)
**Sex (women, %)**	54	52
**Body mass index (kg m^{-2})**	28.26 (5.34)	27.74 (5.72)
**Systolic blood pressure (mm Hg)**	128.59 (17.20)	115.16 (14.04)
**Diastolic blood pressure (mm Hg)**	73.43 (10.05)	74.25 (9.42)
**High density lipoprotein (mg dL^{-1})**	57.28 (18.02)	59.07 (16.77)
**Total cholesterol (mg dL^{-1})**	185.69 (37.22)	184.93 (31.62)
**Triglycerides (mg dL^{-1})**	118.86 (69.97)	107.20 (58.42)
**Current Smoker (n)**	274	166
**Alcohol drinking (g day^{-1})**	10.27 (15.29)	10.78 (15.15)

Supplementary Table 2: Pedigree structure formed by FHS participants.

Pedigree size	Number of pedigrees	Number of individuals
1	-	456
2 -- 9	424	1771
10 -- 19	58	753
20 -- 99	26	777
≥100	3	413

Supplementary Table 3: Enrichment Tests of *cis-* and *trans*-meQTLs for other molecular QTLs.

		***cis*-meQTL**			***trans*-meQTL**		
	**Total SNPs**	**N of the overlap**	**Fold Change**	**P-value**	**N of the overlap**	**Fold Change**	**P-value**
cis-eSNPs FHS (1e-7)	460,536	399,670	1.66	0	84,591	2.33	0
cis-eSNPs Eur-Meta	398,524	356,500	1.71	0	70,810	2.25	0
cis-pQTLs FHS-1000g	12,401	10,176	1.57	0	2,045	2.09	0
pQTLs FHS-1000g	19,942	16,660	1.6	0	4,428	2.81	0
cis-pQTLs NC2016	376	318	1.62	0	53	1.78	1.63E-05
pQTLs NC2016	456	385	1.61	0	68	1.89	1.79E-07
metabolism QTLs	135	111	1.57	8.38E-14	26	2.44	6.52E-06
GWAS Catalog	32,260	19,000	1.13	0	3078	1.21	0

Supplementary Table 4: Multivariable MR results.

Exposure	Outcome	Other CpGs at r2 > 0.5	beta.MV.MR	se.MV.MR	pval.MV.MR	Group.MV.MR
cg01465596	SBP		-28.82	11.53	6.22E-03	chr_1_4
cg25607920	SBP	cg15644324;cg23762517;cg15937073	4.72	10.55	3.27E-01	chr_1_4
cg18187658	HDL		-0.64	0.14	2.41E-06	chr_1_6
cg24250902	HDL		-0.06	0.65	4.61E-01	chr_1_6
cg16517298	HDL		0.39	0.46	1.99E-01	chr_1_6
cg19911850	DBP		-11.15	11.26	1.61E-01	chr_1_7
cg26303165	DBP		1.88	1.86	1.55E-01	chr_1_7
cg00035347	BMI		-0.13	0.08	5.30E-02	chr_10_1
cg10872209	BMI		0.38	0.09	1.19E-05	chr_10_1
cg03493300	CHD		1.80	2.41	2.28E-01	chr_10_1
cg09803321	CHD		-3.41	1.33	5.19E-03	chr_10_1
cg00894378	CHD		-4.71	3.23	7.20E-02	chr_10_1
cg18534077	CHD		3.59	2.09	4.33E-02	chr_10_1
cg09803321	MI		-4.63	1.02	3.12E-06	chr_10_1
cg03493300	MI		0.36	0.84	3.34E-01	chr_10_1
cg00894378	MI		-0.63	0.91	2.43E-01	chr_10_1
cg03493300	SBP		-17.52	0.56	4.38E-216	chr_10_1
cg00894378	SBP		-14.15	1.85	1.08E-14	chr_10_1
cg19610905	HDL		2.86	2.40	1.17E-01	chr_11_1
cg11250194	HDL		-1.83	0.62	1.55E-03	chr_11_1
cg11250194	LDL		-1.18	2.45	3.15E-01	chr_11_1
cg19610905	LDL		15.20	9.08	4.70E-02	chr_11_1
cg11250194	TC		-0.48	3.01	4.37E-01	chr_11_1
cg19610905	TC		17.85	10.00	3.71E-02	chr_11_1
cg11250194	TG		-0.97	4.46	4.14E-01	chr_11_1
cg19610905	TG		-2.52	6.56	3.50E-01	chr_11_1
cg21709803	TG	cg06781209	-0.54	4.03	4.47E-01	chr_11_1
cg00603274	TG		3.34	8.07	3.39E-01	chr_11_1
cg18512352	HDL		1.50	1.20	1.05E-01	chr_11_2
cg09580214	HDL		0.03	0.14	4.12E-01	chr_11_2
cg21033440	DBP	cg24147428	3.82	3.04	1.05E-01	chr_11_3
cg24468199	DBP		23.72	7.03	3.73E-04	chr_11_3
cg16535667	DBP		-9.08	8.18	1.33E-01	chr_11_3
cg24468199	SBP		59.74	2.13	4.07E-174	chr_11_3
cg21033440	SBP		-7.74	3.83	2.17E-02	chr_11_3
cg14842237	SBP		-8.90	1.73	1.33E-07	chr_11_3
cg00211115	SBP		-4.77	3.35	7.73E-02	chr_11_5
cg23478547	SBP		-3.34	1.50	1.28E-02	chr_11_5

cg20608306	TG		-2.02	0.52	5.60E-05	chr_11_6
cg11861562	TG	cg26566898;cg15534755;cg10130564;cg03276022;cg16524733	-0.76	0.27	2.79E-03	chr_11_6
cg26624021	TC		2.08	0.27	2.97E-15	chr_16_2
cg02254551	TC		0.62	0.18	2.81E-04	chr_16_2
cg21433558	MI		1.01	0.94	1.40E-01	chr_17_1
cg21692620	MI		-1.47	0.68	1.52E-02	chr_17_1
cg26162295	HDL		1.79	0.23	3.38E-15	chr_17_5
cg18711369	HDL		-5.31	3.75	7.82E-02	chr_17_5
cg10909506	HDL		3.86	2.85	8.82E-02	chr_17_5
cg00810710	T2D		-1.32	1.49	1.88E-01	chr_17_6
cg26853458	T2D		1.98	1.55	1.01E-01	chr_17_6
cg11023668	BMI	cg01884057;cg04586622	-0.34	0.06	8.26E-10	chr_2_1
cg09505516	BMI		0.40	0.30	8.80E-02	chr_2_1
cg16888658	BMI		-0.05	0.37	4.51E-01	chr_2_1
cg02493740	MI		-0.64	0.83	2.23E-01	chr_2_2
cg23752985	MI		0.55	2.77	4.21E-01	chr_2_2
cg26034919	TG	cg04845466	-1.86	2.99	2.66E-01	chr_2_8
cg22903471	TG		14.08	9.28	6.46E-02	chr_2_8
cg17986701	TG		-3.88	2.53	6.25E-02	chr_20_1
cg00981651	TG		4.70	4.37	1.41E-01	chr_20_1
cg13276631	DBP		24.40	10.84	1.22E-02	chr_3_1
cg04625862	DBP		-11.78	2.61	3.17E-06	chr_3_1
cg13276631	SBP		38.33	7.64	2.58E-07	chr_3_1
cg20734569	SBP		2.93	0.70	1.40E-05	chr_3_1
cg21832537	HDL		-0.51	0.09	1.25E-08	chr_3_3
cg27127091	HDL		-0.37	0.14	4.05E-03	chr_3_3
cg03032677	TG		0.56	0.03	1.77E-75	chr_6_2
cg15474579	TG		-0.59	0.47	1.05E-01	chr_6_2
cg23833896	SBP		-15.23	6.93	1.40E-02	chr_8_6
cg17694851	SBP		19.21	2.81	4.09E-12	chr_8_6
cg13506600	LDL		-1.33	3.32	3.44E-01	chr_9_1
cg24267699	LDL	cg21160290;cg22535403;cg11879188	2.30	3.22	2.37E-01	chr_9_1
cg07241568	MI		10.46	4.30	7.53E-03	chr_9_1
cg24267699	MI		-1.42	1.20	1.18E-01	chr_9_1
cg24267699	TC	cg21160290;cg22535403;cg11879188	3.65	2.03	3.62E-02	chr_9_1
cg13506600	TC		-2.74	2.09	9.54E-02	chr_9_1

Supplementary Table 5: Putatively causal gene expression traits for CVD and CVD risk factors using GTEx eQTLs.

Outcome	Exposure	MR method	Number of SNPs	beta.MR	se.MR	pval.MR
CHD	VAMP8 (all tissues in GTEx)	Inverse variance weighted	3	-0.10	0.03	1.43E-04
MI	LIPA (Adrenal Gland)	Wald ratio	1	0.18	0.03	8.61E-13
MI	LIPA (Pancreas)	Wald ratio	1	0.25	0.04	1.24E-12
MI	LIPA (Thyroid)	Wald ratio	1	0.28	0.04	1.34E-12
MI	LIPA (Colon Transverse)	Wald ratio	1	0.23	0.03	1.34E-12
MI	LIPA (Artery Aorta)	Wald ratio	1	0.33	0.05	1.86E-12
MI	LIPA (Lung)	Wald ratio	1	0.21	0.03	1.86E-12
MI	LIPA (Whole Blood)	Wald ratio	1	0.16	0.02	2.71E-12
MI	LIPA (Spleen)	Wald ratio	1	0.18	0.03	2.71E-12
MI	LIPA (Liver)	Wald ratio	1	0.26	0.04	3.05E-12
MI	LIPA (Skin Sun Exposed Lower leg)	Wald ratio	1	0.40	0.06	3.05E-12
CHD	LIPA (Whole Blood)	Wald ratio	1	0.14	0.02	5.15E-12
CHD	LIPA (Spleen)	Wald ratio	1	0.16	0.02	5.15E-12
CHD	LIPA (Adrenal Gland)	Wald ratio	1	0.15	0.02	8.25E-12
CHD	LIPA (Colon Transverse)	Wald ratio	1	0.20	0.03	1.06E-11
CHD	LIPA (Thyroid)	Wald ratio	1	0.24	0.03	1.06E-11
CHD	LIPA (Skin Sun Exposed Lower leg)	Wald ratio	1	0.35	0.05	2.14E-11
CHD	LIPA (Liver)	Wald ratio	1	0.22	0.03	2.14E-11
CHD	LIPA (Artery Aorta)	Wald ratio	1	0.28	0.04	2.73E-11
CHD	LIPA (Lung)	Wald ratio	1	0.17	0.03	2.73E-11
CHD	LIPA (Pancreas)	Wald ratio	1	0.21	0.03	2.84E-11
DBP	SDCCAG8 (Colon Transverse)	Wald ratio	1	1.03	0.18	6.06E-09
DBP	SDCCAG8 (Cells Transformed fibroblasts)	Wald ratio	1	0.46	0.08	7.96E-09
CHD	VAMP8 (Esophagus Mucosa)	Wald ratio	1	-0.12	0.02	8.72E-09
DBP	SDCCAG8 (Adipose Subcutaneous)	Wald ratio	1	0.55	0.10	1.11E-08
DBP	SDCCAG8 (Whole Blood)	Wald ratio	1	0.52	0.09	2.16E-08
DBP	SDCCAG8 (Esophagus Mucosa)	Wald ratio	1	0.57	0.10	2.41E-08
DBP	SDCCAG8 (Artery Tibial)	Wald ratio	1	0.74	0.13	2.93E-08
DBP	SDCCAG8 (Heart Atrial Appendage)	Wald ratio	1	0.66	0.12	2.93E-08
CHD	VAMP8 (Muscle Skeletal)	Wald ratio	1	-0.18	0.03	4.66E-08
DBP	SDCCAG8 (Brain Cortex)	Wald ratio	1	0.44	0.08	5.03E-08
CHD	AS3MT (Brain Caudate basal ganglia)	Wald ratio	1	0.08	0.02	6.47E-08
DBP	SDCCAG8 (Adipose Visceral Omentum)	Wald ratio	1	0.68	0.13	1.28E-07
DBP	SDCCAG8 (Spleen)	Wald ratio	1	0.37	0.07	1.51E-07

DBP	SDCCAG8 (Nerve Tibial)	Wald ratio	1	0.47	0.09	1.51E-07
DBP	SDCCAG8 (Breast Mammary Tissue)	Wald ratio	1	0.60	0.12	3.13E-07
DBP	SDCCAG8 (Thyroid)	Wald ratio	1	0.81	0.16	5.01E-07
DBP	SDCCAG8 (Artery Aorta)	Wald ratio	1	0.49	0.10	7.58E-07
SBP	SDCCAG8 (Adipose Visceral Omentum)	Wald ratio	1	0.97	0.21	3.29E-06
DBP	SDCCAG8 (Ovary)	Wald ratio	1	0.35	0.08	5.79E-06
DBP	SDCCAG8 (Artery Coronary)	Wald ratio	1	0.45	0.10	5.79E-06
SBP	SDCCAG8 (Esophagus Mucosa)	Wald ratio	1	0.74	0.17	7.56E-06
DBP	SDCCAG8 (Colon Sigmoid)	Wald ratio	1	0.50	0.12	1.76E-05
CHD	AS3MT (Whole Blood)	Wald ratio	1	0.08	0.02	1.82E-05
CHD	AS3MT (Artery Coronary)	Wald ratio	1	0.04	0.01	1.87E-05
CHD	AS3MT (Pituitary)	Wald ratio	1	0.04	0.01	1.87E-05
CHD	AS3MT (Skin Sun Exposed Lower leg)	Wald ratio	1	0.05	0.01	1.87E-05
CHD	AS3MT (Brain Cerebellar Hemisphere)	Wald ratio	1	0.04	0.01	1.87E-05
CHD	AS3MT (Muscle Skeletal)	Wald ratio	1	0.06	0.01	1.87E-05
SBP	SDCCAG8 (Brain Cortex)	Wald ratio	1	0.56	0.13	2.06E-05
CHD	AS3MT (Brain Cerebellum)	Wald ratio	1	0.04	0.01	2.23E-05
CHD	AS3MT (Skin Not Sun Exposed Suprapubic)	Wald ratio	1	0.05	0.01	2.23E-05
CHD	AS3MT (Esophagus Muscularis)	Wald ratio	1	0.06	0.01	2.23E-05
CHD	AS3MT (Esophagus Mucosa)	Wald ratio	1	0.06	0.01	2.23E-05
CHD	AS3MT (Esophagus Gastroesophageal Junction)	Wald ratio	1	0.06	0.01	2.23E-05
CHD	AS3MT (Thyroid)	Wald ratio	1	0.05	0.01	2.23E-05
CHD	VAMP8 (Testis)	Wald ratio	1	0.12	0.03	2.55E-05
CHD	AS3MT (Brain Anterior cingulate cortex BA24)	Wald ratio	1	0.06	0.01	4.13E-05
SBP	SDCCAG8 (Cells Transformed fibroblasts)	Wald ratio	1	0.52	0.13	4.88E-05
SBP	SDCCAG8 (Colon Transverse)	Wald ratio	1	1.14	0.29	7.44E-05
SBP	SDCCAG8 (Artery Tibial)	Wald ratio	1	0.85	0.22	8.94E-05
SBP	SDCCAG8 (Heart Atrial Appendage)	Wald ratio	1	0.75	0.19	8.94E-05
CHD	AS3MT (Ovary)	Wald ratio	1	0.05	0.01	1.20E-04
DBP	SDCCAG8 (Esophagus Muscularis)	Wald ratio	1	0.67	0.17	1.24E-04
SBP	SDCCAG8 (Adipose Subcutaneous)	Wald ratio	1	0.60	0.16	1.25E-04
T2D	LAMC1 (Brain Cerebellum)	Wald ratio	1	-0.05	0.01	1.26E-04
SBP	SDCCAG8 (Artery Coronary)	Wald ratio	1	0.61	0.16	1.38E-04
SBP	SDCCAG8 (Ovary)	Wald ratio	1	0.47	0.12	1.38E-04
SBP	SDCCAG8 (Breast Mammary Tissue)	Wald ratio	1	0.72	0.19	1.49E-04
CHD	AS3MT (Uterus)	Wald ratio	1	0.04	0.01	1.62E-04
SBP	SDCCAG8 (Whole Blood)	Wald ratio	1	0.55	0.15	2.05E-04

T2D	LAMC1 (Artery Tibial)	Wald ratio	1	0.18	0.05	2.46E-04
SBP	SDCCAG8 (Artery Aorta)	Wald ratio	1	0.59	0.16	2.62E-04
SBP	SDCCAG8 (Thyroid)	Wald ratio	1	0.95	0.26	2.78E-04
CHD	AS3MT (Vagina)	Wald ratio	1	0.05	0.01	4.85E-04
T2D	LAMC1 (Whole Blood)	Wald ratio	1	-0.10	0.03	5.37E-04
SBP	SDCCAG8 (Nerve Tibial)	Wald ratio	1	0.49	0.14	7.64E-04
SBP	SDCCAG8 (Spleen)	Wald ratio	1	0.39	0.12	7.64E-04
SBP	SDCCAG8 (Colon Sigmoid)	Wald ratio	1	0.63	0.19	8.73E-04
T2D	LAMC1 (Brain Cerebellar Hemisphere)	Wald ratio	1	-0.07	0.02	9.41E-04

Supplementary Table 6: Gene ontology enrichment results of *cis*-eGenes of *trans*-meQTL hotspots.

Term	Overlap Gene Number	P Value	Genes	Fold Enrichment	FDR (provided by DAVID:https://david.ncifcrf.gov/)
GO:0003676~nucleic acid binding	21	2.92E-10	ZNF208, HKR1, ZNF566, ZNF100, ZFP30, ZNF260, ZNF781, ZNF200, ZNF75A, ZNF793, ZNF333, ZNF177, ZNF585B, ZNF738, ZNF429, ZNF540, ZNF607, ZNF561, ZNF493, ZNF266, ZNF573	5.54	3.31E-07
GO:0006355~regulation of transcription, DNA-templated	24	1.88E-09	ZNF208, HKR1, ZNF566, ZNF100, ZFP30, ZNF260, ZNF200, ZNF781, ZNF75A, ZNF793, ZNF333, ZNF177, ZNF585B, TCF7L1, ZNF738, ZNF429, ZNF492, ZNF540, INO80E, ZNF607, ZNF561, ZNF493, ZNF266, ZNF573	4.19	2.51E-06
GO:0046872~metal ion binding	27	9.42E-09	ZNF208, HKR1, YPEL3, ZNF200, ZNF781, ZNF75A, ZNF177, CISD2, ZNF429, DPEP3, ZNF492, ZNF540, ZNF607, ZNF561, ZNF493, ZNF266, DPEP2, ZNF566, ZNF100, MAT2A, ZFP30, ZNF260, ZNF793, ZNF333, GDPD3, ZNF585B, ZNF573	3.39	1.07E-05
GO:0006351~transcription, DNA-templated	26	1.12E-08	ZNF208, HKR1, E2F4, ZNF200, ZNF781, ZNF75A, ZNF177, TCF7L1, ZNF738, ZNF429, ZNF492, ZNF540, INO80E, ZNF607, ZNF561, ZNF493, ZNF266, ZNF566, ZNF100, ZFP30, ZNF260, ZNF793, ZNF333, ZNF585B, MAPK3, ZNF573	3.49	1.50E-05
GO:0003677~DNA binding	22	4.58E-07	ZNF208, HKR1, ZNF566, SP140L, E2F4, ZFP30, ZNF260, ZNF781, NFKB1, ZNF75A, ZNF333, ZNF177, TCF7L1, ZNF738, ZNF429, ZNF540, ZNF607, TIGD7, NFATC3, ZNF493, ZNF266, ZNF573	3.41	5.19E-04
GO:0003700~transcription factor activity, sequence-specific DNA binding	15	1.13E-05	E2F4, ZFP30, ZNF260, ZNF200, NFKB1, ZNF793, ZNF585B, TCF7L1, ZNF429, ZNF540, ZNF561, ZNF607, NFATC3, ZNF493, ZNF573	4.05	0.012811

Supplementary Table 7: CVD casual CpG in association with smoking and alcohol consumption.

CpG	Traits.MR	CHR.CpG	MAPINFO.CpG	Gene Symbol	Association Direction	Associated Environmental Factor
cg00894378	SBP/DBP/MI/CHD	10	104680152	CNNM2	Negative	Alcohol Drinking
cg00981651	TG	20	44574847	PCIF1	Positive	Smoking
cg01465596	SBP/DBP	1	42381916	HIVEP3	Positive	Smoking
cg02254551	TC	16	56998544	CETP	Negative	Alcohol Drinking
cg03032677	TG	6	36648794	CDKN1A	Negative	Smoking
cg14037218	CHD	1	150522367	ADAMTSL4	Negative	Smoking
cg15474579	TG	6	36645812	CDKN1A	Negative	Smoking
cg15741354	SBP	8	8146129		Positive	Smoking
cg16517298	HDL	1	230413174	GALNT2	Negative	Smoking
cg17986701	TG/HDL	20	44574422	PCIF1	Positive	Smoking
cg18257541	T2D	3	23244062	UBE2E2	Negative	Alcohol Drinking
cg18711369	HDL	17	38081186	ORMDL3	Negative	Alcohol Drinking
cg21692620	MI	17	40835849	CNTNAP1	Positive	Smoking
cg21832537	HDL	3	52553167	STAB1	Negative	Alcohol Drinking
cg22118147	DBP	5	172144013		Positive	Smoking
cg23956648	T2D	3	185479235	IGF2BP2	Negative	Alcohol Drinking
cg24250902	HDL	1	230415547	GALNT2	Negative	Smoking
cg26624021	TC/TG	16	56995739	CETP	Negative	Smoking
