# Supplementary material for: Genome-wide identification of DNA methylation QTLs in whole blood highlights pathways for cardiovascular disease
Source: Nat Commun. 2019 Sep 19;10:4267. doi: 10.1038/s41467-019-12228-z (PMC6753136; doi:10.1038/s41467-019-12228-z)
Supplement: Supplementary file 9 — Reporting Summary [file 41467_2019_12228_MOESM9_ESM.pdf]

nature research

Corresponding author(s): Daniel Levy, Liming Liang, Tianxiao Huan

Last updated by author(s): Jul 10, 2019

Reporting Summary

Nature Research wishes to improve the reproducibility of the work that we publish. This form provides structure for consistency and transparency in reporting. For further information on Nature Research policies, see Authors & Referees and the Editorial Policy Checklist.

Statistics

For all statistical analyses, confirm that the following items are present in the figure legend, table legend, main text, or Methods section.

n/a	Confirmed	
☐	☒	The exact sample size (*n*) for each experimental group/condition, given as a discrete number and unit of measurement
☐	☒	A statement on whether measurements were taken from distinct samples or whether the same sample was measured repeatedly
☐	☒	The statistical test(s) used AND whether they are one- or two-sided<br>*Only common tests should be described solely by name; describe more complex techniques in the Methods section.*
☐	☒	A description of all covariates tested
☐	☒	A description of any assumptions or corrections, such as tests of normality and adjustment for multiple comparisons
☐	☒	A full description of the statistical parameters including central tendency (e.g. means) or other basic estimates (e.g. regression coefficient) AND variation (e.g. standard deviation) or associated estimates of uncertainty (e.g. confidence intervals)
☐	☒	For null hypothesis testing, the test statistic (e.g. *F*, *t*, *r*) with confidence intervals, effect sizes, degrees of freedom and *P* value noted<br>*Give P values as exact values whenever suitable.*
☒	☐	For Bayesian analysis, information on the choice of priors and Markov chain Monte Carlo settings
☒	☐	For hierarchical and complex designs, identification of the appropriate level for tests and full reporting of outcomes
☐	☒	Estimates of effect sizes (e.g. Cohen's *d*, Pearson's *r*), indicating how they were calculated

Our web collection on statistics for biologists contains articles on many of the points above.

Software and code

Policy information about availability of computer code

Data collection	no software used
Data analysis	A custom Java script used for meQTL identification is deposited in xx. All the other algorithms and softwares are used published / established methods and R packages as described in Methods section.

For manuscripts utilizing custom algorithms or software that are central to the research but not yet described in published literature, software must be made available to editors/reviewers. We strongly encourage code deposition in a community repository (e.g. GitHub). See the Nature Research guidelines for submitting code & software for further information.

Data

Policy information about availability of data

All manuscripts must include a data availability statement. This statement should provide the following information, where applicable:

- Accession codes, unique identifiers, or web links for publicly available datasets
- A list of figures that have associated raw data
- A description of any restrictions on data availability

The complete set of DNAm data for FHS participants has been deposited in and is available from dbGaP under study phs00363.001v12.p9. The meQTL resources developed for this study are freely accessible via the NCBI Molecular QTL Browser (https://preview.ncbi.nlm.nih.gov/gap/eqtl/studies/) and via the NCBI ftp site (https://ftp.ncbi.nlm.nih.gov/eqtl/original_submissions/FHS_meQTLs/).

Field-specific reporting

Please select the one below that is the best fit for your research. If you are not sure, read the appropriate sections before making your selection.

☒ Life sciences ☐ Behavioural & social sciences ☐ Ecological, evolutionary & environmental sciences

For a reference copy of the document with all sections, see nature.com/documents/nr-reporting-summary-flat.pdf

Life sciences study design

All studies must disclose on these points even when the disclosure is negative.

Sample size	This study used all available samples (n=4170) in FHS whose DNA methylation data and genotypes (passing quality control) were both available.
Data exclusions	We excluded samples whose DNA methylation data failed quality control as provided in method section.
Replication	The results were successfully replicated in 963 samples in ARIC study and 384 samples in GTP study.
Randomization	Not applicable
Blinding	Not applicable

Reporting for specific materials, systems and methods

We require information from authors about some types of materials, experimental systems and methods used in many studies. Here, indicate whether each material, system or method listed is relevant to your study. If you are not sure if a list item applies to your research, read the appropriate section before selecting a response.

Materials & experimental systems

n/a	Involved in the study	
☒	☐	Antibodies
☒	☐	Eukaryotic cell lines
☒	☐	Palaeontology
☒	☐	Animals and other organisms
☐	☒	Human research participants
☒	☐	Clinical data

Methods

n/a	Involved in the study	
☒	☐	ChIP-seq
☒	☐	Flow cytometry
☒	☐	MRI-based neuroimaging

Human research participants

Policy information about studies involving human research participants

Population characteristics	The population characteristics were provided in Supplementary Table 1.
Recruitment	no applicable
Ethics oversight	The FHS study protocol was approved by Boston Medical Center. The GTP study was approved by Institutional Review Boards of Emory University School of Medicine and Grady Memorial Hospital. The ARIC Study is conducted in four US communities including Forsyth County, North Carolina; Jackson, Mississippi; suburbs of Minneapolis, Minnesota; and Washington County, Maryland. The ARIC study protocol was approved by the institutional review board of each participating university.

Note that full information on the approval of the study protocol must also be provided in the manuscript.
