# Supplementary material for: Genome-wide identification of DNA methylation QTLs in whole blood highlights pathways for cardiovascular disease
Source: Nat Commun. 2019 Sep 19;10:4267. doi: 10.1038/s41467-019-12228-z (PMC6753136; doi:10.1038/s41467-019-12228-z)
Supplement: Supplementary file 10 — Description of Additional Supplementary Files [file 41467_2019_12228_MOESM10_ESM.pdf]

Title: Supplementary Data 1:
Description: CpGs with h2 >0.1.

Title: Supplementary Data 2:
Description: CpGs with household effects >0.1.

Title: Supplementary Data 3:
Description: Mendelian randomization results of causal CpGs for CVD and CVD risk factors.

Title: Supplementary Data 4:
Description: Pathway analysis of cis-meQTLs for 92 CVD related CpGs by FUMA.

Title: Supplementary Data 5:
Description: Putatively causal gene expression traits for CVD and CVD risk factors using FHS blood eQTLs.

Title: Supplementary Data 6:
Description: trans-meQTL hotspots.

Title: Supplementary Data 7:
Description: Linking GWAS p values with peak cis-meQTLs for each CVD causal CpG.
